# Implementing Leading antimicrobial stewardship practices in United States hospitals – A qualitative study

**DOI:** 10.1017/ash.2022.101

**Published:** 2022-05-16

**Authors:** Salome Chitavi, Mike Kohut, Barbara Braun, Meghann Adams, David Hyun

## Abstract

**Background:** In May 2018, The Joint Commission, The Pew Charitable Trusts, and the CDC cosponsored a meeting of experts who identified 6 evidence-based leading practices that antimicrobial stewardship programs (ASPs) should be doing beyond having basic infrastructure for improving antibiotic prescribing. The Joint Commission Department of Research working with external experts in 2020 conducted a prevalence study to assess what proportion of Joint Commission-accredited hospitals had implemented the 6 leading practices identified (results presented at SHEA Spring 2021). In this qualitative study, we collected information about how hospitals implemented ASP leading practices to identify facilitators and barriers to implementation among diverse hospitals. **Methods:** We conducted in-depth telephone interviews with a subset of ASP leaders from hospitals that participated in the 2020 prevalence study. We used purposive sampling to select 30 hospitals from 288 hospitals based on leading practices implemented, hospital size, and system membership. An experienced qualitative researcher (M.K.) not previously affiliated with the Joint Commission interviewed all participants using a semistructured interview guide. The framework method of analysis was used to review and organize data. We used the constant comparative approach to ensure that factors were not missed. Each transcript was reviewed by at least 2 researchers who compared coded findings in group discussion sessions. Two researchers independently identified key factors and combined findings following discussion and review. We focused on super factors that are relevant to implementing multiple leading practices. **Results:** ASP leaders from 30 hospitals were interviewed. Participating hospitals were evenly distributed across hospital size (10 small, 10 medium, 10 large) and membership in a health system (16 system, 14 nonsystem). At least 14, (46.7%) interviewees had pharmacist in their title; 11 (36.7%) had pharmacist-antimicrobial stewardship; and 5 (16.6%) had other titles (eg, infection preventionist). Super factors included ASP team capacity, ID expertise, having a physician champion, relationships with clinicians and relevant departments, structure of electronic health records, adequate software, and information technology resources. Small and rural nonsystem hospitals often lacked resources related to ID expertise, dedicated staff, and software tools, whereas hospitals that belong to a system benefit from centralized ID expertise and technical infrastructure provided. **Conclusions:** Specific factors related to personnel, relationships and IT resources have an outsized impact on implementing multiple leading antimicrobial stewardship practices in hospitals. Hospital ASPs could benefit by targeting resources toward these areas.

**Funding:** None

**Disclosures:** None

